# Piles or Hemorrhoids

**Published:** 1875-09

**Authors:** 


### PILES OR HEMORRHOIDS.

Few, besides physicians, know how common this disease is. In fact, there is intense ignorance concerning it, in every way. Sowe will endeavor to throw light upon it, from time to time. First, then, piles are usually tumors in and about the lower extremity of the intestinal canal. But wherever they exist, there is congestion or inflammation. Sometimes they are merely enlarged veins. They are ordinarily of slow growth. It has always been a difficult matter to cure them. Men and women have spent fortunes in their efforts to rid themselves of this fearful infliction. They are caused by bad living, in most cases. Whatever weakens the body; whatever tends to render the liver torpid, the skin inactive, the kidneys ineffective,—will induce piles. Every form of weakening excess, every act of dissipation encourages this disease. Bad water or bad other drink, bad air and bad food will induce it, unfailingly. What shall we do for relief and what for cure? Clearly, we must lessen the congestion or the inflammation, if the inflammatory stage has been reached. This accomplished, pain ceases. To overcome the pain is the main thing. We are positive that the local application of cold will reduce the congestion and the inflammation, and will thus overcome the pain and burning. We are not aware that cold can be applied to these parts, by any method, except through the use of Dr. Mattson's pile-cone. This does the work effectively. Pain ceases almost magically, and the brain—always more or less excited in these cases—is quickly soothed into calmness and sweet repose. Of course reasonable living is demanded to ensure a complete cure, but the cone is certain to relieve the pain and discomfort.

---

DIPPING.—A Providence, R. I., tobacconist has put up a sign in his shop window, giving notice that he sells "Snuff for dipping," and the *Journal* of that city waxes irate over the introduction of this filthy habit. Strange as it may appear, "dipping" is confined almost entirely to women, and is practised to a much greater extent than would be imagined. Of course it would be almost useless to indicate to a woman capable of "dipping" that she is a fool, but perhaps some who are acquiring the habit "just for fun," or because their companions urge them, or for some equally frivolous reason, might be saved from the debasement if convinced of what is a widely-known fact, that persistence will make their voices harsh, give them pinched faces and hollow eyes, and shortly transform the most attractive girl into a repulsive, prematurely aged creature. It does really seem as though there are enough healthful and decent pleasure and stimulants at the command of both sexes and all classes to satisfy even the most exacting, and prevent their emulating savages and "low-down" whites of the South.

